# La grossesse extra-utérine dans une région semi-rurale en Afrique: Aspects épidémiologiques, cliniques et thérapeutiques à propos d'une série de 74 cas traités à l'Hôpital de District de Sangmelima au Sud-Cameroun

**Published:** 2012-11-30

**Authors:** Bruno Kenfack, Michel Noubom, Adamo Bongoe, Faustin Atemkeng Tsatedem, Modeste Ngono, Georges Nguefack Tsague, Emile Mboudou

**Affiliations:** 1 Département des Sciences Biomédicales, Faculté des Sciences Université de Dschang, Cameroun. Hôpital de District de Dschang, Cameroun; 2 Hôpital Régional d'Edéa, Cameroun; 3 Hôpital de District de Logbaba, Douala, Cameroun; 4 Faculté de Médecine et des Sciences Biomédicales, Université de Yaoundé I, Cameroun

**Keywords:** Grossesse extra-utérine, epidémiologie, clinique, traitement, Cameroun, ectopic-pregnancy, epidemiology, clinic, treatment, Cameroon

## Abstract

La grossesse extra-utérine (GEU) constitue une cause fréquente de morbidité et parfois de mortalité chez les femmes en âge de procréation. Son étiologie n'est pas clairement précisée. Son tableau clinique est polymorphe et ses méthodes thérapeutiques très diversifiées. C'est dans le but d’étudier les aspects épidémiologiques cliniques et thérapeutiques dans une zone rurale à ressources limitées d'Afrique que ce travail a été réalisé. Il s'agit d'une étude descriptive transversale sur une durée de trois ans, portant sur 74 cas de GEU traités à l'Hôpital de District de Sangmelima. Le matériel utilisé était constitué d'une fiche anonyme de collecte des données, des dossiers du malade, et du registre opératoire. Au cours de la période d’étude, 2142 naissances vivantes ont été enregistrées, soit un taux de GEU de 3,45%. Les femmes non mariées et celles ayant les antécédents d'IST étaient les plus atteintes. Le délai moyen entre le début des symptômes et l'admission était de132h. L’âge gestationnel moyen au moment du diagnostic était de 8,14 semaines. Le diagnostic était clinique dans 61% des cas. L'annexe controlatérale était cliniquement normale dans 53% des cas. Le traitement était chirurgical d'emblée chez 97% des cas. Aucun décès n'a été observé. La GEU est fréquente dans cette zone rurale, les malades consultent à un stade tardif, le diagnostic est surtout clinique, et le traitement chirurgical par laparotomie.

## Aux éditeurs du Journal Panafricain de Médecine

La grossesse extra-utérine (GEU) constitue une cause fréquente de morbidité et parfois de mortalité chez les femmes en âge de procréation. Son étiologie reste incertaine, mais on note une association avec les infections pelviennes et certaines techniques de traitement de l'infertilité [2, 3]. Son diagnostic est souvent difficile, et elle est l'une des rares pathologies don't la prise en charge est très diversifiée [3, 4]. C'est dans le but d’étudier ses aspects que ce travail a été entrepris à Sangmelima au Sud-Cameroun, qui est aussi situé en zone reconnue d'hypofertilité [5].

Il s'agit d'une étude descriptive transversale sur une durée de trois ans, de Juillet 2005 à Juin 2008, à l'Hôpital de District de Sangmelima dans le Sud-Cameroun, sur un total de 74 patientes recrutées de façon consécutive. Le matériel utilisé était constitué des dossiers de malades, d'une fiche anonyme de collecte des données et du registre des comptes rendus opératoires. Le diagnostic était clinique ou échographique, avec une sonde sus pubienne de 3,5Mhz. Parfois l'association des ultrasons au dosage qualitatif des βHCG était nécessaire. Le traitement était d'emblé chirurgical en cas de rupture avec hémopéritoine ou d'un hématosalpinx supérieur à 3 centimètres. Le traitement médical était réservé uniquement aux GEU pauci ou asymptomatiques, sans hémopéritoine. Pendant l'opération, nous avons considéré comme annexe pathologique une occlusion tubaire, un aspect de tuba errecta, des nodosités intra-tubaires à la palpation, une localisation sous adhérentielle de la trompe ou un antécédent d'annexectomie.

**Figure 1:**
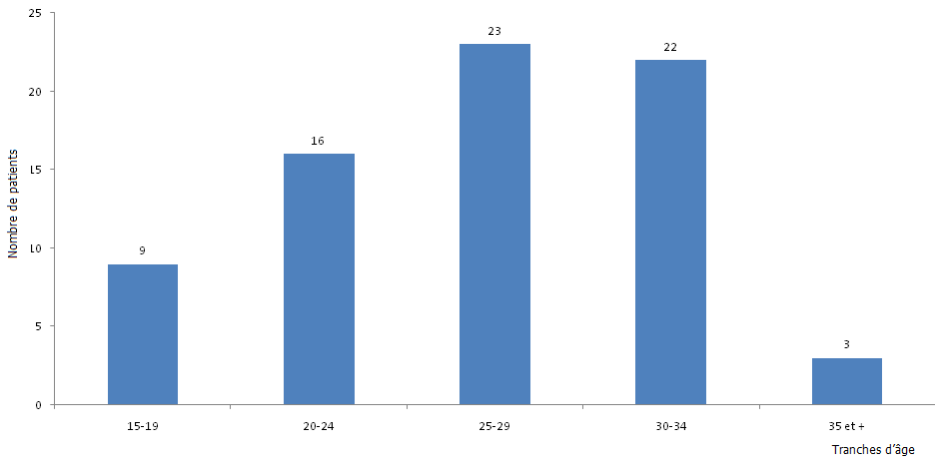
Répartition par tranches d’âge de cinq ans de 74 patientes prises en charge pour grossesse extra-utérine à l'Hôpital de District de Sangmelima de Juillet 2005 à Juin 2008

En trois ans, nous avons enregistré 74 cas de GEU pour 2142 naissances vivantes, soit un taux hospitalier de 3,45%. L’âge des patientes était compris entre 15 et 42 ans, avec une moyenne de 26,46 ± 5,42. La Figure 1 illustre la distribution en fonction des tranches d’âge. Les adolescentes représentaient à elles seules 12,33% des cas. Le Tableau 1 résume les caractéristiques sociales. Le délai moyen entre le début des symptômes et l'admission était de 132h. Le délai entre la dernière grossesse et la GEU en cours variait de 7 à 180 mois, avec une moyenne de 60 ±44 mois. L'histoire d'infertilité était retrouvée chez 68,92% de cas. Les nullipares et les primipares étaient les plus représentées, avec 46 cas au total, soit 62,16%. L’âge gestationnel moyen au moment du diagnostic était de 8,14 semaines, et le nombre moyen d'enfants vivants par femme était de 1,3. Le diagnostic était clinique dans la majorité des cas avec 60,81%, suivi de l’échographie 36,49%. L'ampoule tubaire était la zone la plus touchée, avec 68,06%, et la GEU était rompue dans 69 cas, soit 92%. Le volume moyen de l'hémopéritoine était de 1291 ± 850ml. Les adhérences pelviennes étaient retrouvées chez 74% des cas, et l'annexe controlatérale était cliniquement normale dans 52,8% des cas. Le traitement était chirurgical d'emblée chez 72 patientes, soit 97%. Quinze cas (20,5%) ont été transfusés. Le geste opératoire le plus réalisé était radical (salpingectomie) dans 89% de cas. Aucun cas de complication opératoire ni de mortalité n'a été observé.

**Table 1:** Caractéristiques sociodémographiques et cliniques de 74 patientes prises en charge pour grossesse extra-utérine à l'Hôpital de District de Sangmelima de Juillet 2005 à Juin 2008

Caractéristiques		Nombre	Pourcentage
**Etat civil**			
	Mariée	29	39,19
	Célibataire/concubinage	42	56,76
	Autres	3	4,05
**Profession**			
	Cultivatrice	29	39,19
	Elève/étudiante	14	18,92
	Salariée	10	13,51
	Autres	11	14,86
	Sans	10	13,51
**Ethnie**			
	Beti	57	77,03
	Autres	17	22,97
**Niveau d'instruction**			
	Sans	15	20,27
	Primaire	30	40,54
	Secondaire et plus	29	39,19
**Religion**			
	Chrétienne	68	91,89
	Autres	6	8.1
**Signes et symptômes**			
	Douleurs pelviennes	60	81,08
	Saignement vaginal	40	54,05
	Aménorrhée	69	93,24
**Antécédents**			
	IST (infection sexuellement transmissible)	39	52,70
	Infertilité > 1 an	51	68,92
	GEU	3	4,05
**Issue la précédente grossesse (N = 52)**			
	Accouchement normal	38	51,35
	Avortement/GEU	14	18,92

GEU: Grossesse extra-utérine

Nous avons observé une incidence hospitalière de 3,45%, chiffre très élevé par rapport à celui des pays développés qui varie entre 1% et 2% [2]. Ce chiffre reste au dessus de 1,1% obtenus par KOUAM et al [6] au CHU de Yaoundé. L'incidence élevée de notre étude s'expliquerait par l'hypofertilité déjà objectivée de cette région du pays [5]. Il est bien établi que la GEU est plus fréquente chez les femmes ayant une histoire d'infertilité [2, 7]. L’âge moyen des patientes était de 26,46 ans, inférieur à celui de DOHBIT et al [8] qui était de 29 ans. La région du Sud-Cameroun où a été réalisée notre étude est parmi celles où la sexualité est précoce. En effet, à 15 ans, 50,5% des adolescentes dans notre région d’étude ont déjà eu des rapports sexuels [5]. Le délai moyen entre la dernière grossesse et la GEU en cours était de 60 mois. D'après la littérature, la GEU survient surtout chez les femmes qui ont un antécédent d'infertilité [1]. Dans notre série, 68,92% avaient un antécédent d'infertilité.

Le diagnostic était clinique dans 60,80% de cas. La prédominance de la méthode clinique dans le diagnostic de la GEU est fréquente dans les études réalisées dans les pays en voie de développement [6, 8], où les malades consultent non seulement à un âge avancé de la grossesse, mais aussi très tardivement après le début des symptômes. Le volume moyen de l'hémopéritoine était de 1291±850ml, justifiant la prépondérance de la méthode clinique de diagnostique qui se faisait pour la plupart, par simple paracentèse ou culdocentèse. Les adhérences pelviennes étaient retrouvées chez 74% des cas. Il est bien établi que ces adhérences résultent souvent des infections pelviennes chroniques et sont source de GEU et d'infertilité [1]. En effet 53,4% des cas dans notre série avaient des antécédents d'IST. L'annexe controlatérale était cliniquement normale dans 52,8% des cas. Nous n'avons pas retrouvé les données concernant l’état macroscopique de l'annexe controlatérale dans la littérature, pourtant ce paramètre nous semble important dans pronostic de fertilité ultérieure. Les consultations tardives des patientes, associées aux pertes sanguines élevées justifieraient la prédominance du traitement chirurgical, réalisé dans 97% de cas. Comme dans d'autres études [4, 6], aucun cas de complication opératoire ni de mortalité n'a été observé. Si la prise en charge est immédiate une fois le diagnostic établi, la GEU est rarement mortelle. Dans notre série, tous les cas étaient pris en charge en urgence sans exigence financière ou matérielle préalable.

## Conclusion

La GEU est très fréquente à Sangmelima, avec une incidence hospitalière de 3,45 pour 100 naissances vivantes. Le facteur de risque le plus retrouvé est l'antécédent d'IST. La majorité des patientes arrivent en état de rupture tubaire, avec pour conséquence un traitement chirurgical radical. Les efforts doivent être faits pour amener les patientes à adopter un comportement sexuel responsable et à consulter tôt en cas de signes évoquant une IST ou une GEU.
